# RIP3-mediated necroptosis is regulated by inter-filament assembly of RIP homotypic interaction motif

**DOI:** 10.1038/s41418-020-0598-9

**Published:** 2020-07-31

**Authors:** Hong Hu, Xialian Wu, Guoxiang Wu, Ning Nan, Jing Zhang, Xinxin Zhu, Yu Zhang, Zhaoqian Shu, Jia Liu, Xiaoyan Liu, Junxia Lu, Huayi Wang

**Affiliations:** 1 School of Life Science and Technology, ShanghaiTech University, 393 Middle Huaxia Road, Pudong, Shanghai 201210 China; 2 Chinese Academy of Sciences Center for Excellence in Molecular Cell Science, Shanghai Institute of Biochemistry and Cell Biology, 320 Yueyang Road, Shanghai, 200031 China; 3 University of Chinese Academy of Sciences, 100864 Beijing, China

**Keywords:** Kinases, Cell biology, Protein aggregation

## Abstract

Necroptosis is mediated by signaling complexes called necrosomes, which contain receptor-interacting protein 3 (RIP3) and upstream effectors, such as RIP1. In necrosomes, the RIP homotypic interaction motif (RHIM) of RIP3 and RIP1 forms amyloidal complex. But how the amyloidal necrosomes control RIP3 activation and cell necroptosis has not been determined. Here, we showed that RIP3 amyloid fibrils could further assemble into large fibrillar networks which presents as cellular puncta during necroptosis. A viral RHIM-containing necroptosis inhibitor M45 could form heteroamyloid with RIP3 in cells and prevent RIP3 puncta formation and cell necroptosis. We characterized mutual antagonism between RIP3–RHIM and M45–RHIM in necroptosis regulation, which was caused by distinct inter-filament interactions in RIP3, M45 amyloids revealed with atomic force microscopy. Moreover, double mutations Asn464 and Met468 in RIP3–RHIM to Asp disrupted RIP3 kinase-dependent necroptosis. While the mutant RIP3(N464D/M468D) could form amyloid as wild type upon necroptosis induction. Based on these results, we propose that RIP3 amyloid formation is required but not sufficient in necroptosis signaling, the ordered inter-filament assembly of RIP3 is critical in RIP3 amyloid mediated kinase activation and cell necroptosis.

## Introduction

Necroptosis is a type of programmed necrosis induced by some stress signals related to injury or infection, such as the tumor necrosis factor (TNF) family of cytokines, interferons (IFNs), or lipopolysaccharide [1–4]. Necroptosis execution is mainly controlled by receptor-interacting protein 3 (RIP3) kinase and its substrate mixed-lineage kinase domain-like (MLKL) [5–7]. In TNF-induced necroptosis, the RIP3 activation is mediated by RIP1, which interacts with RIP3 through its RIP homotypic interaction motif (RHIM) domains, forming a specific signaling complex named the necrosome [5–7]. In addition to RIP1 and RIP3, only TIR-domain-containing adapter-inducing interferon-β (TRIF) and DNA-dependent activator of interferon regulatory factors (DAI) have been identified as RHIM-containing proteins in the human genome [8, 9]. TRIF is a cytosolic adapter of Toll-like receptors, and DAI is a sensor of cytoplasmic viral RNA [10, 11]. Both TRIF and DAI can trigger necroptosis through RHIM-dependent recruitment of RIP1 and RIP3 (or RIP3 only when RIP1 is absent) [12–17]. In addition, mammalian RHIMs such as RIP1/3 can form amyloid in vitro and in necrotic cells. Mutants of the conserved residues in the RHIM of RIP1/3 have shown defects in fibril formation and mediating cell necrosis [18, 19]. Thus, RHIM-dependent RIP1/RIP3 amyloid structures are required for necroptosis signal propagation.

RHIM-containing necroptosis regulators have been found in viruses [20, 21]. M45 from murine cytomegalovirus (MCMV) has been reported to inhibit the virus-induced DAI-RIP3 necroptosis pathway [22]. In addition, the M45 homolog ICP6 from herpes simplex virus type 1 inhibits TNF-induced necroptosis in human cells but can induce mouse cell necroptosis [23–26]. M45 and ICP6 are large proteins with over 1000 amino acids consisting of an N-terminal RHIM domain and a C-terminal ribonucleotide reductase (RNR) large subunit (R1)—homology domain [27]. Interestingly, when the M45–RHIM is changed to an ICP6-RHIM, the ICP6-RHIM-containing M45 gains the ability to induce necroptosis in mouse cells [23]. This suggests that these different RHIM domains perform distinct functions by either promoting or inhibiting cell necroptosis. Thus, elucidating how the necroptosis-inhibiting factor M45 blocks necroptosis through its RHIM domain would provide critical insights into the RHIM-dependent necroptosis signaling.

In this study, we found that the accurate inhibitory peptide of the viral necroptosis inhibitor M45 is coincided with M45–RHIM. M45–RHIM binds with RIP3 to form heteroamyloids in cells upon necroptosis induction, and block RIP3 amyloids further assembly into cellular puncta. M45–RHIM and RIP3–RHIM can form fibrils with different morphology in electron microscopy (EM). The atomic force microscopy (AFM) results showed that the morphological differences were caused by the distinct inter-filament interactions, which suggests the structure based the mutual antagonism between RIP3–RHIM and M45–RHIM. In line with that, RIP3 mutant which disrupted the proper inter-filament interactions could not induce necroptosis. These data suggested the ordered inter-filament assembly of RIP3–RHIM into the high-ordered RIP3 amyloid networks as cellular puncta is a critical step of necroptosis signaling, which could be blocked by specific mutations of RIP3 or via the formation of neutralized M45–RIP3 hetero-RHIM amyloids.

## Materials and methods

### Reagents and antibodies

z-VAD and SMAC mimetic compound were used as described previously [24]. Recombinant TNF and IFN-γ were purified in our lab. Necrostatin-1 (HY-15760) and AP20187 (HY-13992) are bought from MCE. Anti-ICP6 polyclonal antibodies were raised in rabbits using *E. coli*-expressed His-tagged ICP6 (1–177 aa). The following antibodies were used in this study: anti-Flag (F3165), anti-Myc (SAB4700448), and anti-mRIP3 (R4277) (Sigma); anti-RIP (D94C12), anti-phospho-RIP (65746S), and anti-hRIP3 (13526S) (Cell Signaling Technology); anti-Phospho-RIP3 (ab209384) and anti-FKBP (ab108420) (Abcam); and anti-actin (PM053-7) (MBL).

### Cell survival assay

The cells were seeded into 96-well plates and allowed to grow for about 12 h at the following density: L929 cells and NIH-3T3 cells, 5000 cells/well; HeLa cells, 6000 cells/well; HT-29 human colon cancer cells, 8000 cells/well. The cells then were treated as indicates (the details were showed in the figures and figure legends). And the cell survival was measured using the CellTiter-Glo Luminescent Cell Viability Assay kit according to the manufacturer’s instructions. Luminescence was recorded with an EnSpire Multimode Plate Reader from PerkinElmer.

### Cell culture and stable cell lines

HEK293T cells, HeLa cells, NIH-3T3 cells, HT-29 human colon cancer cells, and L929 mouse fibroblast cells were obtained from cell bank of CAS (Shanghai). HeLa-RIP3 cells were established as previously described [5, 24, 28]. HT-29 (RIP1-KO) and L929 (RIP1-KO) cells were generated by using the CRISPR/Cas9 editing technique. The KO cells were determined by the sequencing of targeted loci. All cells were cultured in DMEM/HIGH with L-glutamine, without sodium pyruvate (HyClone). All media were supplemented with 10% Foundation™ FBS (Gemini) and 100 units/ml penicillin/streptomycin (HyClone). All cells were cultured at 37 °C with 5% CO2 and tested to be mycoplasma-negative by the standard PCR method. Cells were transfected using Lipofectamine 2000 (Invitrogen), Lipofectamine 3000 (Invitrogen), or EZ transfection (Shanghai Life-iLab Biotech Co., Ltd) according to the manufacturer’s instructions.

### Plasmids and molecular cloning

ICP6 and M45 cDNA was PCR-amplified from a reverse-transcribed cDNA library. Full-length or mutated cDNAs of ICP6 and M45 were cloned into the lentiviral vector pCDH-CMV-MCS-EF1-copGFP (Addgene) using standard PCR and cloning methods. The WT and mutated RIP1 and RIP3 cDNAs were also cloned into the modified lentiviral vector pCDH-CMV-MCS-EF1-copRFP. All plasmids were verified by DNA sequencing. The details of the primer sequences are available upon request.

### Lentivirus preparation and infection

For lentivirus production, HEK293T cells were transfected with lentiviral vectors (pCDH-CMV-MCS-EF1-copGFP/copRFP) and virus packing plasmids (psPAX2 and pMD2.G, Addgene) by using EZ transfection reagents (Shanghai Life-iLab Biotech Co., Ltd). The virus-containing medium was harvested 48 h later and added to the cells as indicated, with 10 μg/ml polybrene. The infection medium was changed with fresh medium 24 h later.

### CRISPR/Cas9

The Cas9-target sites are as follows: human RIP1: 5′-GCTCGGGCGCCATGTAGTAG-3′ and mouse RIP1: 5′-GAAAGGAAGGATAATCGTGG-3′ and 5′-CACCGCTGGAGGTATGGTTCAGCA-3′. The Cas9-target sites were cloned into the PX330 (Addgene) vector. All plasmids were verified by DNA sequencing. The details of the primer sequences are available upon request.

### Immunoprecipitation and immunoblotting

Cells infected with the indicated lentivirus were cultured on 10 cm dishes and grown to confluence. Cells treated as indicated were washed once with DPBS and harvested by scraping and centrifugation at 1000 × *g* for 3 min. The harvested cells were washed with DPBS and lysed for 30 min on ice in lysis buffer containing 25 mM Hepes-NaOH (pH 7.5), 150 mM NaCl, 1% Triton, 10% glycerol, and complete protease inhibitor (Roche) and phosphatase inhibitor (Roche) cocktails. The cell lysates were then centrifuged at a top speed of 12,000 × *g* for 30 min at 4 °C. The soluble fraction was collected, and the protein concentration was determined by a Bradford assay. For direct immunoblot analysis, the samples were subjected to SDS-PAGE and detected using antibodies, as indicated. For immunoprecipitation, 1 mg of extracted protein in lysis buffer was immunoprecipitated overnight with anti-Flag or anti-Myc magnetic beads (Bimake) at 4 °C. After incubation, the beads were washed three times with lysis buffer, then directly boiled in 1X SDS loading buffer and subjected to immunoblot analysis.

### In vitro kinase assay

HEK293T cells were transfected with Flag tagged M45 or RIP3, respectively. After 24 h, RIP3 with or without M45 was immunopurified with anti-Flag magnetic beads (Bimake) at 4 °C overnight. The Flag beads were washed four times with kinase buffer (50 mM HEPES [pH 7.5], 10 mM MgCl_2_, 50 mM NaCl, 0.02% BSA, 150 mM ATP, and 1 mM DTT).Then kinase buffer with the GSK872 (10 μm) was added and incubated at 30 °C for 20 min. After that, 1 μg purified recombinant MLKL was added and reacted at 30 °C for 30 min. Finally, the reaction mixtures were subjected to western blotting analysis.

### Protein expression and purification

The recombinant proteins of MLKL produced as described in previous work [28]. Human wild-type and mutant RIP3 (418–518 aa) with 6×His at the N-terminus was expressed in *E. coli* Rosetta (DE3) cells and induced with 0.8 mM isopropyl b-d-thiogalactoside (Sangon Biotech) at 37 °C for 5 h. The RIP3 peptides were recovered from inclusion bodies by dissolving 20 ml of buffer containing 6 M guanidine hydrochloride, 50 mM Tris-HCl (pH 8.0), and 300 mM NaCl. The denatured RIP3 peptides were purified by Ni-NTA beads 6FF (Smart-Life Science) and then dialyzed with 1 L of pure water at 4 °C. The harvested RIP3 precipitates were dissolved in 25% (v/v) acetic acid solution and further purified by reverse phase high-performance liquid chromatography run on a Waters HPLC machine (2545) at room temperature with a linear gradient of 30–70% aqueous-organic solvent over 10 min at 20 ml/min using an XBridge® Peptide BEH C18 column (130 Åpore, 5.0 μm beads, 19 × 100 mm column, Waters). The aqueous phase consisted of a buffer of Milli-Q H_2_O with 0.05% TFA and an organic solvent of ACN with 0.05% TFA. The purified protein was flash-frozen in liquid nitrogen and dried at −80 °C.

### Analysis of stability of intracellular RIP3 aggregation in pellet fraction

HT-29 or Hela cells infected with the indicated lentivirus were treated with T/S/Z for 6 or 8 h. Then, the cells were washed in DPBS and pelleted by centrifugation at 3000 × *g* for 10 min. The cells were resuspended in 5× volume of buffer A (20 mM HEPES, pH 7.4, 40 mM KCl, 1.5 mM MgCl2, 1 mM EDTA, 250 mM sucrose, complete protease inhibitor (Roche) and phosphatase inhibitor (Roche) cocktails), and homogenized by passing through a 30 G needle 50 times. After centrifugation at 20,000 × *g* for 12 min, the supernatant was harvested and recentrifuged at 100,000 × *g* for 90 min with BECKMA’s ultracentrifuge and the supernatants were harvested as S100. The pellets were resuspended in the same volume of buffer A as P100, for stability assessment of the endogenous M45/RIP3 aggregation. The P100 pellets were resuspended in the same volume of buffer A supplemented with 4 M urea, 8 M urea, or 150 mM NaOH and incubated at room temperature for 30 min, then centrifuged at 100,000 × *g* for 1 h and the pellet was harvested resuspended in the same volume of buffer A as P100. All centrifugations were performed at 4 °C unless otherwise indicated. The samples of S100 and P100 were subjected to western blot analysis with RIP3 or M45 antibodies.

### Immunofluorescence staining and confocal microscopy

HeLa-RIP3 cells infected with the indicated lentivirus were plated on coverslips overnight and treated as indicated. The cells were washed once with PBS and fixed with freshly prepared 4% paraformaldehyde in PBS for 15 min. After permeabilization with 0.1% Triton X-100 in PBS and blockade of nonspecific binding with 4% FBS in PBS, the cells were incubated overnight at 4 °C with primary antibody in PBS. After washing with PBS, the cells were incubated for 60 min with Alexa Fluor 633-conjugated goat anti-mouse IgG (Invitrogen). The nuclei were stained with DAPI (Southern Biotech). All images were captured and processed using identical settings on a Zeiss LSM 710 laser scanning confocal microscope with a ×60 oil objective. Duplicate cultures were performed, and similar results were obtained in at least three independent experiments.

### Fibril preparation

M45 (52–71 aa) was chemically synthesized by Guoping Pharmaceutical Co. Ltd (China). RIP3 (418–518 aa) and RIP3 (418–518 aa, N464D/M468D) were prepared in the laboratory, as described in the “Materials and Methods” section. Lyophilized protein powder of RIP3 or M45 was dissolved in 6 M guanidine hydrochloride buffer (pH 7.5) or 1,1,1,3,3,3-hexafluoro-2-propanol, and fibrils were formed by dilution in Milli-Q water. The hetero RIP3–M45 fibrils were generated by dilution of an equimolar mixture of denatured RIP3 and M45 peptides in Milli-Q water. The samples were incubated at room temperature for ~3 days and then dialyzed for ~3 days in Milli-Q water at room temperature with Spectra/por®6 Dialysis Membranes (MWCO 3.5 K, W 45 mm, Diam 29 mm; BBI Life Science). The water was changed three times at 6-h intervals during dialysis.

### Transmission electron microscopy

One drop of fibril solution was deposited onto a 300-mesh carbon-coated grid (Beijing Zhongjingkeyi Technology) and was kept on the grid for 5 min. The grid was washed twice using Milli-Q water and then stained with 2% uranyl acetate in water (w/v) for negative staining. Finally, excess liquid was blotted off and allowed to air dry. Electron micrographs were recorded with an electron microscope (FEI) Talos L120C (120 kV).

### X-ray fibril diffraction

Fibril pellets were collected by ultracentrifugation at 55,000 rpm and 25 °C for 1 h (Optima Max-TL, BECKMAN COULTER). The fibril pellet was then mounted in a loop and exposed to Cu-*κ* radiation from a Bruker D8 VENTURE X-ray generator at a wavelength of 0.154184 nm, and the distance was 50 mm from the source. Data were collected at room temperature for 1 min on a Bruker D8 VENTURE imaging plate detector.

### Atomic force microscopy

A fibril suspension was deposited onto the freshly cleaved mica surface and incubated for 5 min at room temperature. The mica sheets were subsequently rinsed twice with Milli-Q water to remove the unbound substance. All imaging was performed under air conditions in tapping mode using antimony (n)-doped Si cantilevers with a constant force of 40 N/m (model RTESPA-300, Bruker, USA) at ~300 kHz on a Dimension Icon AFM with a Bruker Nanoscope V controller (Digital Instruments, Goleta, CA, USA). The images were recorded at a scan rate of 1 Hz. Fibril heights were measured by subtracting the average baseline from the highest peak, generating an accurate reading of the fibril diameters.

### Thioflavin T (ThT) fluorescence binding

A ThT binding assay was performed using an EnSight™ Multimode Plate Reader by PerkinElmer. ThT was used at a concentration of 46.4 μm in ThT binding buffer. Seeds were prepared by sonicating the diluted fibril solution using a 2 mm sonication probe with a pulse of 3 s work and 3 s rest for 20 cycles at 25% amplitude while keeping the sample on ice. The peptide stock solution was added to ThT binding buffer with or without a 1 μm concentration of the indicated seeds to a final concentration of 10 μm prior to fluorescence measurement. The data were collected by measuring the fluorescence intensity (485 nm) every 2 min for ~2 h and were analyzed with Origin software.

### Quantification and statistical analysis

**Fig. 1:**
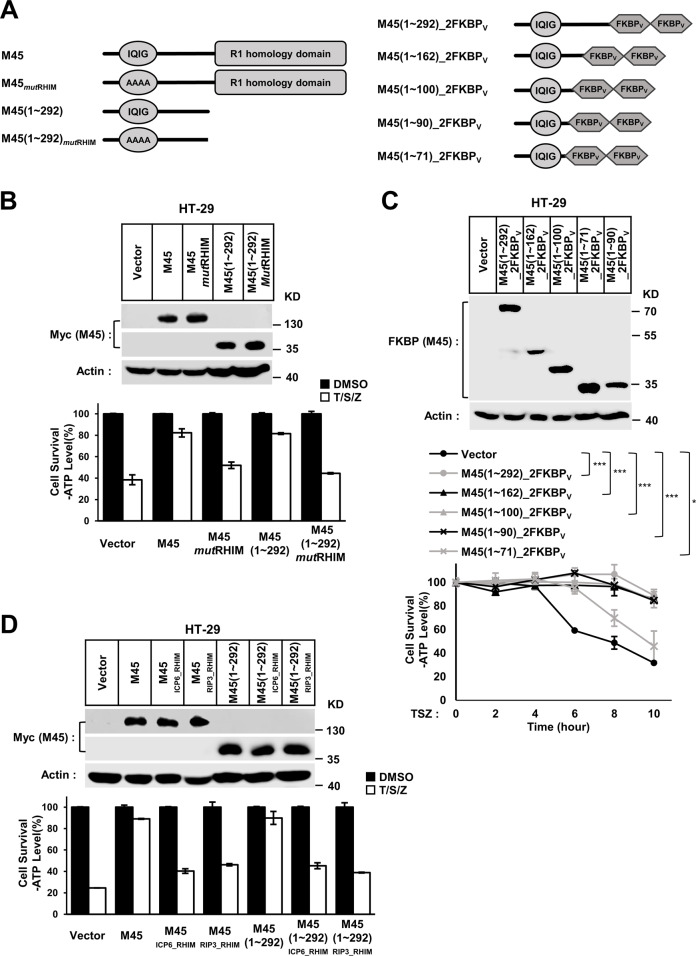
Mapping the exact inhibitory region of M45. **a** Schematic representation of full-length M45 and M45 (1–292 aa) with or without RHIM mutations used in these studies (left). Schematic representation of M45 truncations with tandem FKBPv domain at its C-terminus (right). The four conserved residues (IQIG of M45) of RHIM or their alanine mutations destroy RHIM function are indicated. **b** Cell necroptosis is blocked by full-length or N-terminal portion of M45. The cells with indicated lentivirus infection were treated with T/S/Z for human HT-29 for 10 h. The number of surviving cells was determined by measuring ATP levels using CellTiter-Glo kit (lower). The data are represented as the mean ± standard deviation (SD) of duplicate wells. Similar results were obtained from at least three independent experiments. T TNF-a, S Smac mimetic, Z z-VAD. The final concentrations of 10 ng/ml TNF-a, 100 nM Smac mimetic, and 20 μm z-VAD were used. Identical concentrations of these necrosis-inducing agents were used in subsequent experiments unless otherwise stated. The untreated cells were harvested and whole-cell extracts were prepared and normalized to the same concentration. Aliquots of 20 μg whole-cell lysates were subjected to SDS-PAGE followed by western blot analysis of M45 (Myc) and β-Actin which is shown as a loading control (upper). **c** Mapping the M45 functional region. HT-29 cells were infected with lentiviruses encoding M45 truncations treated with T/S/Z for the indicated time. The number of surviving cells was determined by measuring ATP levels (lower). The expression level of M45 truncations was measured by western blot analysis (upper). Statistical significance was determined using two-way ANOVA followed by a Dunnett post hoc test. Significance between samples is indicated in the figures as follows: ns not significant; *P < 0.05, **P < 0.01, and ***P < 0.001. **d** The chimaeric M45 containing ICP6 or RIP3–RHIM could barely block TNF-induced cell necrosis. The schematic diagram of chimaeric M45 is shown in Fig. S1A. HT-29 cells with indicated lentivirus infection were treated with T/S/Z for 10 h. The number of surviving cells was determined by measuring ATP levels (lower). The data are represented as the mean ± SD of duplicate wells. The expression level of chimaeric M45 was measured by western blot analysis (upper).

**Fig. 2:**
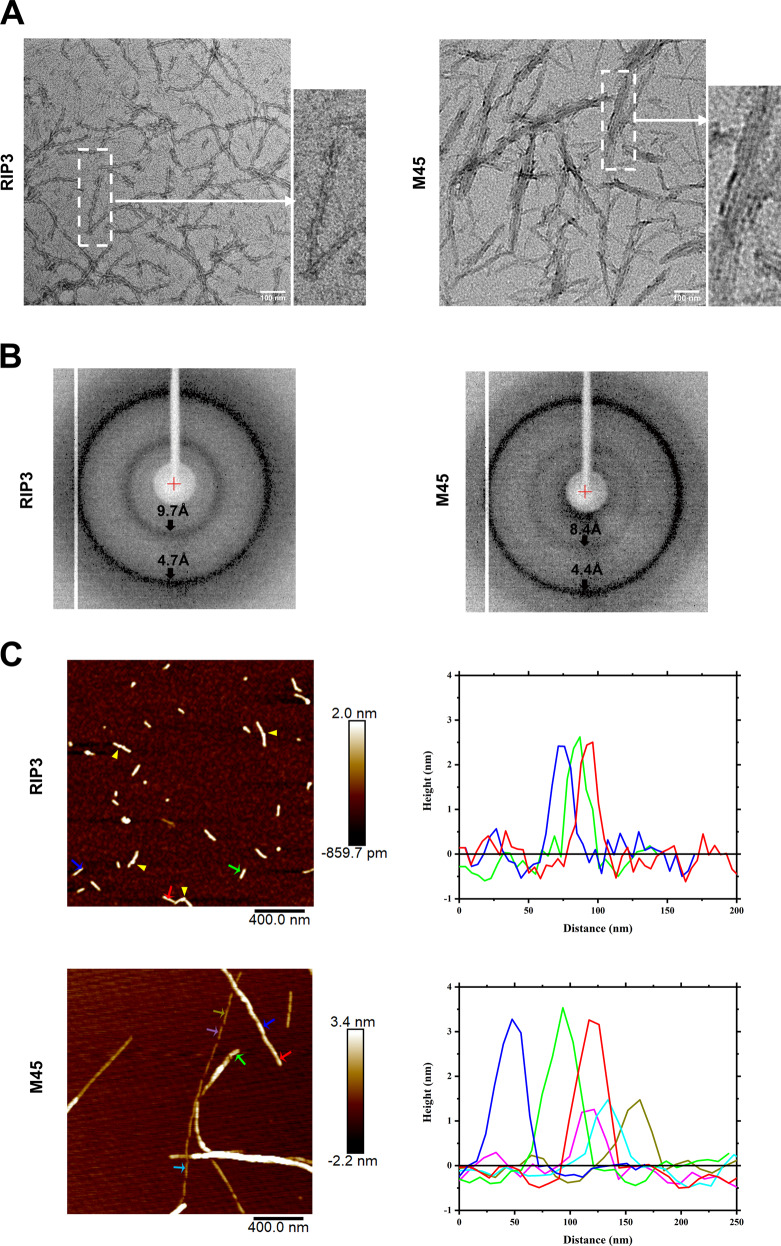
EM and AFM analysis of M45–RHIM and RIP3–RHIM amyloid fibrils. **a** Negative-stained EM images of RIP3 fibrils (left) and M45 fibrils (right). The amyloid fibrils assembled from 0.1 mg/ml human RIP3 (418–518 aa) or 0.2 mg/ml M45 (52–71 aa) as described in “Materials and Methods” (scale bar 100 nm). **b** The X-ray diffraction images of RIP3 fibrils (left) and M45 fibrils (right). The arrow indicated equatorial and meridional reflection of a cross-β-sheet structure arrangement. For RIP3 fibrils, the equatorial and meridional reflection were at about ~9.7 and 4.7 Å, respectively; while the equatorial and meridional reflection of M45 fibrils were at ~8.4 and 4.4 Å, respectively, different from RIP3. **c** AFM images and the height profile of RIP3 fibril and M45 fibril. The yellow triangle indicates the head-tail connection in RIP3 fibrils. The arrows indicate the positions to obtain the height profile data. RIP3 fibrils (upper) have a uniform height of 2.3 ± 0.3 nm; M45 fibrils (lower) composed of two kinds of height. One is 1.6 ± 0.3 nm and the other is 3.2 ± 0.3 nm.

**Fig. 3:**
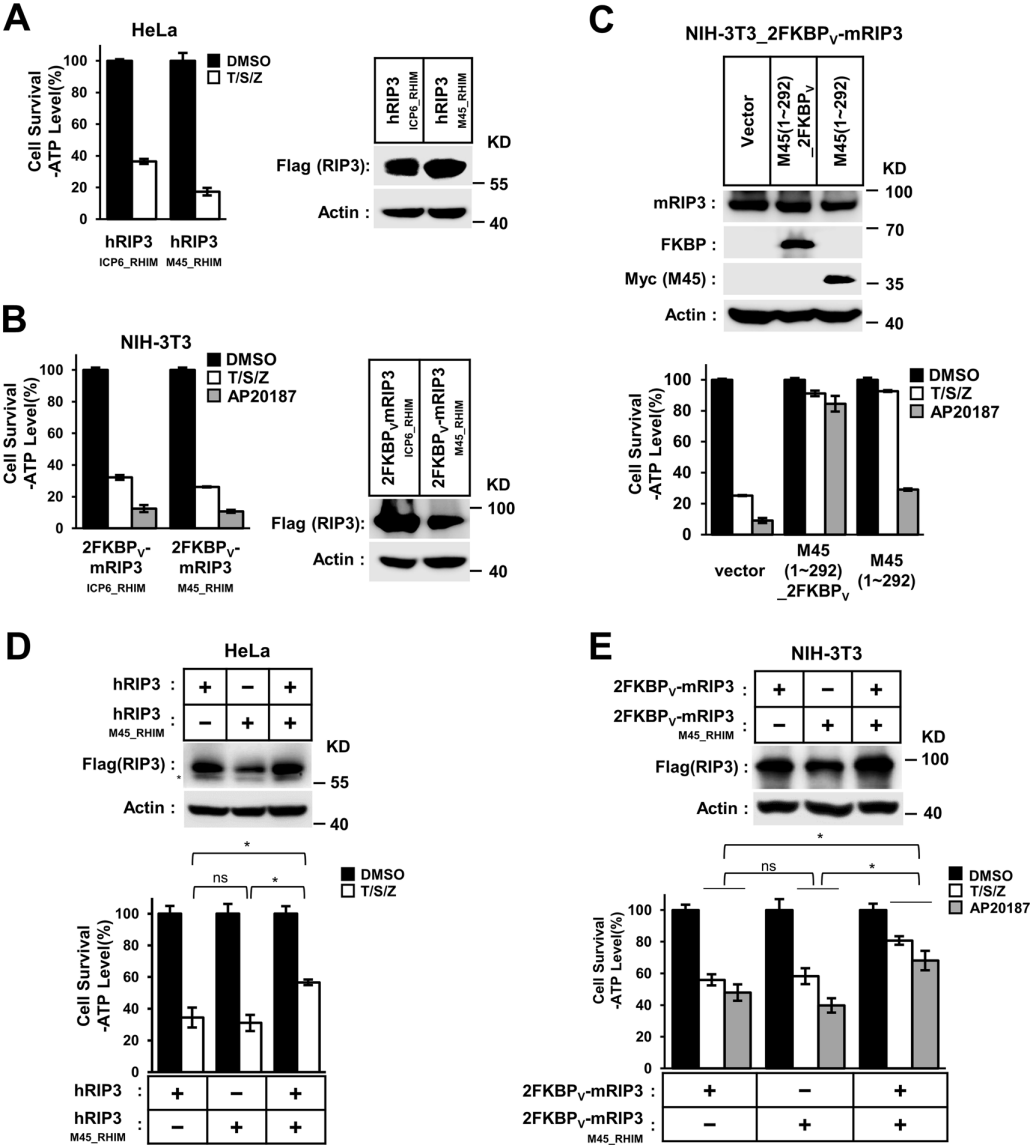
Domain swapping investigation of M45–RHIM function. **a**, **b** RIP3 chimeras gain the sensitivity to necroptosis induction. HeLa (**a**) or NIH-3T3 (**b**) cells with indicated lentivirus infection were treated with T/S/Z or AP20187 for 10 h. The number of surviving cells was determined by measuring ATP levels (left). The data are presented as the mean ± SD of duplicate wells. The expression level of chimaeric RIP3 was measured by western blot analysis (right). **c** NIH-3T3 cells with expression of tandem FKBP_V_ fused mouse RIP3 (NIH-3T3_2FKBP_V_-mRIP3) were infected with empty viruses (vector) or lentiviruses encoding M45 (1–292 aa) or tandem FKBP_V_ fused M45 (1–292 aa)_V_, then treated with T/S/Z or AP20187 for 10 h. The number of surviving cells was determined by measuring ATP levels (lower). Data are represented as mean ± SD of duplicate wells. The whole-cell lysates were analyzed by western blotting with the indicated antibodies (upper). HeLa (**d**) or NIH-3T3 (**e**) cells were transiently transfected with wild-type or chimaeric RIP3 as indicated. Eighteen hours later, the cells were treated as indicated for 10 h. Then cell viability was determined by measuring ATP levels (lower). The data are represented as the mean ± SD of triplicate wells. Statistical significance was determined using one-way ANOVA followed by a Tukey post hoc test; ns not significant and *P < 0.05. The protein levels were measured by western blot analysis (upper).

All data are shown as the mean ± standard deviation of duplicate wells or triplicate wells, and similar results were obtained from at least three independent experiments. Statistical analysis was carried out with GraphPad Prism 7, GraphPad Software Inc. (San Diego, CA). In cell survival assays, the ATP level of all of control wells in each type of cell was used to check data distribution using a Kolmogorov–Smirnov test. And the results showed that the data from cell death assays fitted a normal distribution. Statistical significance for the cell death assays was then determined using two-way ANOVA (Figs. 1c, 2f, and S3C) followed by Dunnett’s post hoc test when comparing each group to the vehicle control and one-way ANOVA (Fig. 3d, e) or two-way ANOVA (Fig. S3D) followed by a Tukey post hoc test when comparing all pairs of groups. In all tests, the significance of differences between samples is indicated in the figures as follows: ns not significant; *P < 0.05, **P < 0.01, and ***P < 0.001.

## Results

### The core necroptosis-inhibitory peptide of M45, Gly45-Thr90

The MCMV protein M45 was discovered as a negative regulator of premature cell death during infection of endothelial cells and macrophages [29]. Subsequent studies found that its function is dependent on RHIM-mediated blockage of necroptosis pathways [22, 30, 31]. M45 consists of an N-terminal RHIM domain and a C-terminal RNR large subunit (R1)-homology domain [27]. To characterize the specific effects of the M45–RHIM and R1 domains in greater detail, we prepared expression vectors that encoded Myc-tagged full-length and truncated forms of M45 with or without mutations of four alanine substitutions disrupting their RHIM domains (Fig. 1a, left). We found that truncated M45 (1–292 aa) can block necroptosis in HT-29 cells and that the RHIM mutants lose this ability (Fig. 1b). This suggests that the RHIM but not the R1 domain of M45 is necessary for this function. We also tested M45 (1–292 aa) in HeLa cells containing stably expressed RIP3 (HeLa-RIP3) or mouse L929 cells. In both kinds of cells, M45 (1–292 aa) had an ability similar to that of full-length M45 to block TNF-induced necroptosis (Fig. S1B, c). This suggests that this RHIM-containing region is sufficient to block cell necrosis. Interestingly, although the viral homolog ICP6 has domain structures similar to those of M45 (Fig. S1A, left), it needs both the RHIM and R1 domains to block TNF-induced necroptosis in HT-29 cells (Fig. S1D). It has been reported that ICP6- or chimaeric M45_ICP6_RHIM_-induced mouse cell necroptosis is dependent on the oligomerization of the R1 domain of ICP6 or M45_ICP6_RHIM_ [23]. Chemical (AP20187)-induced oligomerization of the tandem FKBPv domain could restore the ability of ICP6 lacking the R1 domain to induce mouse cell necroptosis [23]. Although the R1 domain is not necessary for M45 to block cell necroptosis, we wondered whether the oligomerization state of R1 contributes to the blocking ability of M45. Thus, we replaced the R1 domain of M45 with tandem FKBP_V_ (Fig. 1a, right). We found that AP20187-induced oligomerization did not improve the blocking ability of the M45 N-terminal region (1–292 aa) (Fig. S1E). This suggests that M45 function is completely dependent on its N-terminal RHIM-containing region. To map the core region responsible for blocking necroptosis, we constructed several Myc-tagged M45 N-terminal constructs (Figs. 1a, right, and  S2A). Our data showed that the M45 peptides 1–71 aa, considerably block cell necroptosis and that other M45 peptides, such as 1–90 aa and 45–162 aa, almost completely block necroptosis (Figs. 1c and S2C). Besides that, M45 may block cell necroptosis through inhibiting RIP3 activation. We found the RIP3 autophosphorylation induced by necrotic induction was blocked by these M45 truncations (Fig. S2D). Therefore, M45 N-terminal region (Gly45-Thr90) containing the RHIM core is sufficient to block RIP3 activation and cell necroptosis. To confirm the unique function of M45–RHIM, we replaced the RHIM core region of M45 (M45_52–71) with that of ICP6 (ICP6_65–83) or RIP3 (RIP3_448–468) (Figs. S1A, right, and S2B). We found that neither M45_ICP6_RHIM_ nor M45_RIP3_RHIM_ could block necroptosis, as observed for wild-type M45 (Fig. 1d). Consisted with that, RIP3 autophosphorylation was not blocked by these M45 chimaeras (Fig. S2E). Interestingly, these M45 chimaeras showed retarded necroptosis to a certain extent (Fig. S2F). This may be because these M45 chimaeras could also bind to RIP1/3 to interfere with necrosis signaling. All of these findings suggest that the M45–RHIM is unique and sufficient to block cell necrosis.

### M45–RHIM and RIP3–RHIM form different amyloid fibrils revealed by EM and AFM analysis

Recent studies have reported that the RHIM of RIP1 and RIP3 can form functional amyloid fibrils, which is required for RIP3 activation [18, 19]. Our data suggested that M45–RHIM is sufficient to block necroptosis. We then hypothesized that M45–RHIM may differ from RIP1/3-RHIM, in that it may not form amyloid fibrils. So that it will bind and prevent RIP1/3-RHIM from forming functional amyloid fibrils. To test this hypothesis, we first synthesized RHIM core region peptides of M45 (52–71) and tested amyloid fibril formation in vitro. Negatively stained EM images of M45 and RIP3 peptides revealed that M45–RHIM can form fibrillar polymers like RIP3–RHIM (Fig. 2a). X-ray powder diffraction of the samples yielded the characteristic “cross-β” diffraction pattern, which confirmed that these are amyloid fibrils (Fig. 2b). Interestingly, the M45 fibrils in EM were morphologically different from the RIP3 fibrils. The RIP3 fibrils were single-stranded with fibrillar segments connecting together (Fig. 2a, left panel), while M45 formed bundles of fibrils with various widths (Fig. 2a, right panel). Thus, RIP3 and M45 amyloid polymers have different architectures. Further analysis of diluted fibrils by AFM revealed that the RIP3 peptides tend to form uniformly dispersed short fibrils with similar fibril heights. And the head-tail connection of fibrils can be easily detected (Fig. 2c, upper panel). On the other hand, M45 peptides form single fibrils or fibrillar bundles, and the height of the fibrils showed clear variations (Fig. 2c, lower panel). This shows the inter-filament interactions of RIP3 and M45 amyloids differ.

### Mutual antagonism between RIP3–RHIM and M45–RHIM

**Fig. 4:**
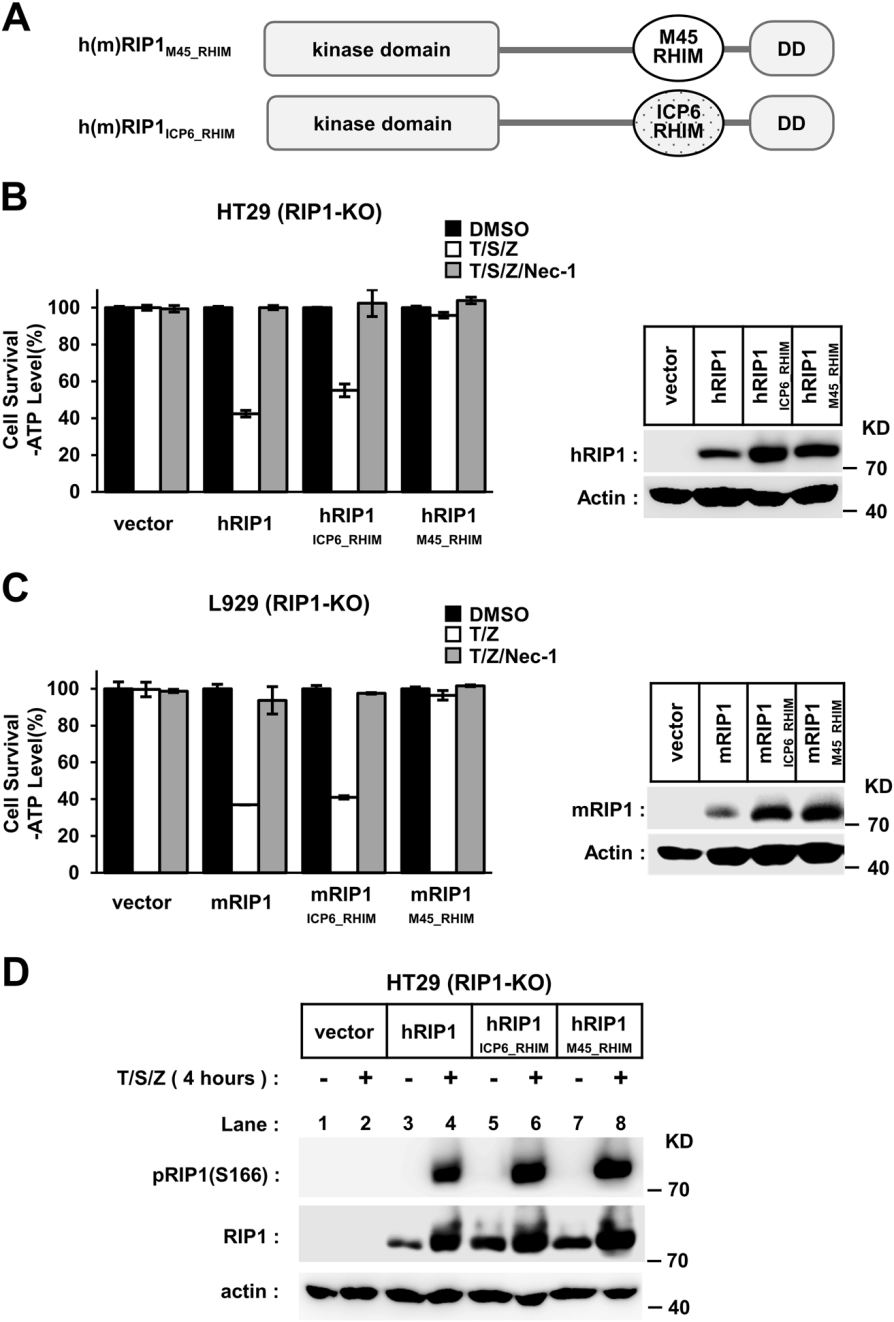
RIP1M45_RHIM blocks necroptosis signal transduction downstream of RIP1 kinase activation. **a** Schematic representation of chimaeric RIP1-containing M45/ICP6-RHIM. **b**, **c** RIP1 chimeras with M45–RHIM could not restore the sensitivity to necrotic stimuli in RIP1 knockout cells. RIP1 knockout HT-29 (**b**) or L929 (**c**) cells with indicated lentivirus infection were treated with indicated stimuli for 10 h (**b**) or 4 h (**c**). The final concentrations of 10 μm RIP1 inhibitor necrostatin-1 (Nec-1) were used to block necrosis. The number of surviving cells was determined by measuring ATP levels (left). The data are presented as the mean ± SD of duplicate wells. The RIP1 expression level was measured by western blot analysis (right). **d** TNF-induced RIP1 kinase activation is not affected in chimaeric RIP1. RIP1-KO HT-29 cells with indicated lentivirus infection were treated with the indicated stimuli for 4 h. The cells were harvested and analyzed by western blotting using antibodies as indicated.

Considering that M45–RHIM can assemble into fibrils with a unique morphology, a possible cause of M45 inhibitory function could be proposed. This kind of fibril may have a “closed-state” conformation that is detrimental to RIP1/RIP3–RHIM-dependent necrosis signal transduction. To determine whether M45–RHIM itself is inhibitory during RIP3 activation, we replaced the RIP3–RHIM core region with that of M45 or ICP6 (as a control) (Figs. S2B and S3A, left). We found that HeLa cells transfected with RIP3_M45_RHIM_ or RIP3_ICP6_RHIM_ remained sensitive to necroptosis (Fig. 3a). Similarly, in NIH-3T3 cells transfected with chimaeric 2FKBP_V_-mRIP3_M45_RHIM_ or 2FKBP_V_-mRIP3_ICP6_RHIM_, both TNF-induced RIP1 activation and AP20187-induced RIP3_M45–RHIM_ or RIP3_ICP6_RHIM_ assembly cause cell necroptosis (Fig. 3b). We also replaced the RIP3–RHIM core region with M45 N-terminal peptides (1–71 aa and 1–90 aa) and made several RIP3_M45_1–71_ and RIP3_M45_1–90_ chimaeric constructs (Fig. S3A, right). Similar results were obtained with those RIP3 chimaeras. Cells with either RIP3_M45_1–71_ or RIP3_M45_1–90_ expression were sensitive to necroptosis induction (Fig. S3B, C). These data suggest that M45–RHIM is functionally equivalent to RIP3–RHIM in RIP3 activation. We also tested the function of chimaeric RIP1 with the core region of M45–RHIM or ICP6-RHIM (Fig. 4a). We found that in RIP1 knockout HT-29 and L929 cells, the expression of wild-type RIP1 or RIP1_ICP6_RHIM_ but not RIP1_M45_RHIM_ could restore sensitivity to necrotic stimuli (Fig. 4b, c). These data indicate that the M45–RHIM core region could replace RIP3–RHIM but not RIP1-RHIM during necroptosis signaling. We then tested whether M45–RHIM blocked RIP1 kinase activation when embedded in RIP1. We used the RIP1 phosphor antibody (pS161) to measure RIP1 kinase autophosphorylation. We found that TNF-induced kinase activation of this chimaeric RIP1 was not affected (Fig. 4d). This suggests that RIP1_M45_RHIM_ blocks necroptosis signal transduction downstream of RIP1 kinase activation.

The activation of either chimaeric RIP1_M45_RHIM_ or chimaeric RIP3_M45_RHIM_ was not affected, suggesting that M45–RHIM alone had no inhibitory effects. Therefore, RIP1_M45_RHIM_ may block necroptosis signaling through M45–RHIM (in RIP1_M45_RHIM_) binding to RIP3–RHIM. To test whether M45–RIP3 binding has an inhibitory effect on RIP3 activation, we transfected M45 (1–292) or M45 (1–292)_2FKBP_V_ into mouse NIH-3T3 cells expressing 2FKBP_V_-mRIP3, in which RIP1-independent cell necroptosis could be induced chemically (AP20187). We found that M45 (1–292) could efficiently block TNF-induced RIP1-dependent necroptosis but could only attenuate cell death caused by AP20187-induced RIP3 self-assembly (Figs. 3c and S3D), while M45 (1–292)_2FKBP_V_ effectively blocked both the RIP1-dependent and RIP1-independent necroptosis pathways (Fig. 3c). The different effects of M45 (1–292) and M45 (1–292)_2FKBP_V_ on RIP1-independent necroptosis indicated that the spontaneous binding of M45 and RIP3 is rare and that AP20187 promotes the binding of M45 and RIP3 in M45 (1–292)_2FKBP_V_ cells to improve M45 effects on RIP3. These data showed that M45 could directly block RIP3 self-assembly-induced cell necrosis when it binds to RIP3 and suggested that RIP1 may function to help M45 bind to RIP3 in the RIP1-containing complex. To confirm the cooperative ability of M45–RHIM and RIP3–RHIM to block necroptosis, we exchanged the RHIM between RIP3 and M45 and found that chimaeric M45_RIP3_RHIM_ barely blocked necroptosis in wild-type RIP3-expressing cells but efficiently blocked necroptosis in RIP3_M45_RHIM_-expressing cells (Fig. S3E, F). Interestingly, although either wild-type RIP3 or chimaeric RIP3_M45_RHIM_ alone could mediate necroptosis, these components interfere with each other in response to necroptosis induction when transiently expressed in HeLa or NIH-3T3 cells (Fig. 3d, e). These experiments reinforce the idea that the mutual antagonism between RIP3–RHIM and M45–RHIM in necroptosis regulation.

### M45–RHIM prevents RIP3 fibrils from self-assembling into cellular puncta

**Fig. 5:**
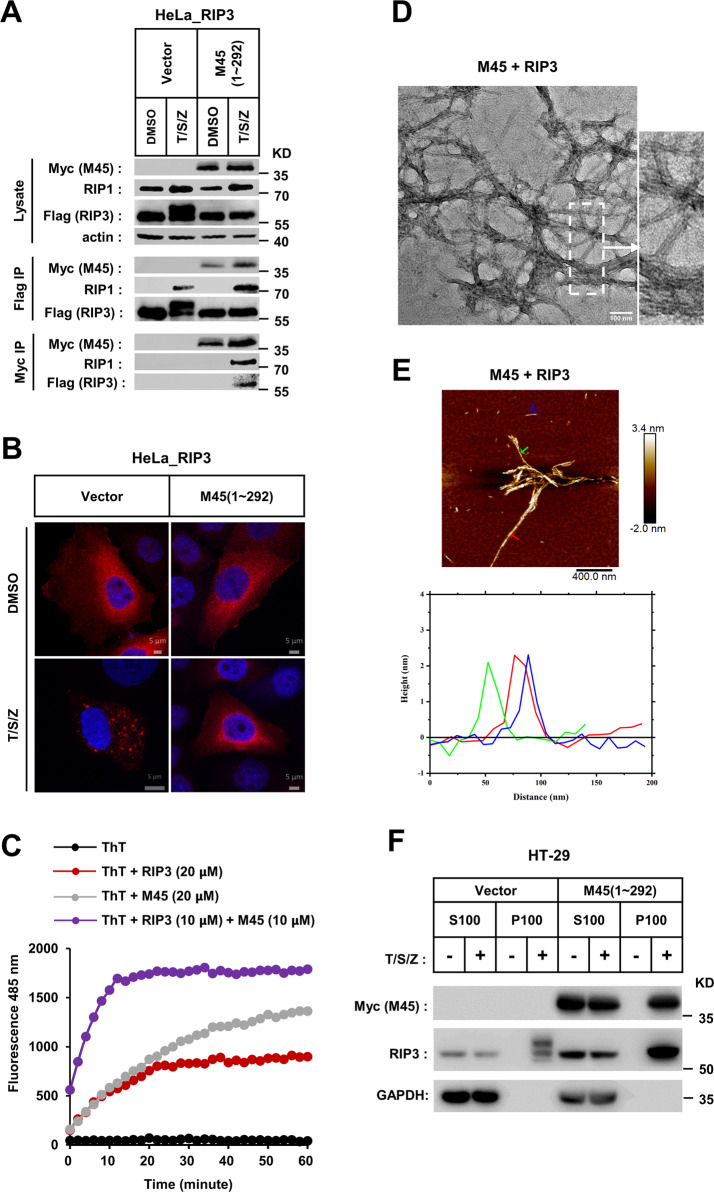
M45–RHIM prevents RIP3 fibrils from self-assembling into cellular puncta. **a** M45 do not repress RIP1-RIP3 binding in necrosome. RIP3-HeLa cells were infected with empty virus (vector) or lentiviral virus encoding Myc-tagged M45 (1–292) in the presence of T/S/Z for 6 h. Whole-cell lysates were subjected to immunoprecipitation with anti-Flag M2 beads or anti-Myc beads. The total cell lysates and immunoprecipitates were immunoblotted with the indicated antibodies. **b** M45 can block TNF-induced cellular RIP3 puncta formation. RIP3-HeLa cells infected with empty virus (vector) or lentiviral virus encoding Myc-tagged M45 (1–292) were treated as the indicated for 6 h. The distribution of RIP3 (red) was detected by immunofluorescence as described in “Materials and Methods.” The scale bar represents 5 μm. **c** Assembly profile of different peptides (M45 + RIP3) (purple); M45 (gray); RIP3 (red) monitored by ThT fluorescence binding assay as described in “Materials and Methods.” **d** EM image of M45 + RIP3 heteromeric amyloid fibrils formed by dilution equimolar mixed RIP3 and M45 stock solution into water as described in “Materials and Methods” (scale bar 100 nm). **e** AFM images and the height profile of RIP3–M45 amyloid fibrils. The arrow indicated the position to obtain the height profile data. Hetero RIP3–M45 fibril with a uniform height of 2.1 ± 0.3 nm. **f** Analysis of intracellular RIP3 aggregation. HT-29 cells infected with the indicated lentivirus were treated with T/S/Z for 8 h. The cells were harvested and homogenized, then separated into the indicated fractions as described in “Materials and Methods.” The distribution of RIP3 and M45 in indicated fractions was analyzed by western blot. S supernatant, P precipitate.

Extensive studies have previously established that self-assembly of RHIMs is responsible for RIP1-RIP3 necrosome formation [18, 19, 32, 33]. When necroptosis was induced by TNF, RIP1 was activated, and RIP3 was then recruited to form the necrosome. This could be revealed by monitoring necroptosis signal-dependent enhancement of RIP1-RIP3 binding. This necroptosis-specific assembly of RIP1-RIP3 is regarded as the critical step of RIP3 necrosome initiation [5, 28]. Our data show that M45–RHIM can functionally replace RIP3–RHIM to induce cell necrosis, but it can block cell necrosis when heterologously expressed. This finding impelled us to check whether M45 could affect RHIM-dependent RIP1-RIP3 binding during necroptosis signaling. We found that although RIP3 kinase autophosphorylation (RIP3 band shift) was blocked by M45 (1–292) expression, the necroptosis induction triggered by RIP1-RIP3 binding was not affected (Fig. 5a). We also tested M45–RIP1 and M45–RIP3 binding by precipitation of Myc-tagged M45 (1–292). We found that the binding of both M45–RIP1 and M45–RIP3 was weak in untreated cells but enhanced upon necroptosis induction (Fig. 5a). This result indicates that the activated RIP1 induced by TNF will recruit both RIP3 and M45 to the necrosome and promote more M45–RIP3 binding. This could explain why M45 functions less efficiently in the RIP1-independent necroptosis pathway (Fig. 3c). Thus, in the TNF-induced necroptosis pathway, M45 does not suppress RIP1-RIP3 necrosome initiation but invades this necrosis signaling complex. We next checked whether M45 played a role in the steps of RIP3 necrosome maturation, during which RIP3 self-assembly was observed as puncta in necrotic cells [28]. The RIP3 puncta could be stained by the classical amyloid fibril dye ThT, suggesting that the RIP3 puncta were composed of RIP3 amyloids [18]. As expected, discrete puncta of RIP3 were detected in control cells transfected with empty vector. However, in M45 (1–292)-expressing cells, RIP3 remained uniformly diffuse throughout the cytosol upon necroptosis induction (Fig. 5b). This suggests that although M45 does not prevent RIP1-RIP3 binding in necrosomes, it can block RIP3 amyloids from forming cellular puncta.

### M45 block RIP3 activation through forming M45–RIP3 heteroamyloid

The cellular RIP3 puncta are large protein clusters of RIP3 amyloids. So that M45–RHIM will block RIP3 puncta assembly before or after RIP3–RHIM amyloid formation. We first tested whether M45 prevents RIP3–RHIM from forming amyloid fibrils in vitro. We mixed denatured M45 and RIP3 peptides and then diluted them in ThT binding buffer to measure fibrillar polymerization during refolding. Compared with RIP3 or M45 peptides alone, we found that the ThT fluorescence increase of the M45–RIP3 peptide mixture was faster, and the fluorescence intensity was also much stronger (Fig. 5c). This suggests that RIP3–RHIM and M45–RHIM may prefer to form heteroamyloid fibrils when mixed together. Interestingly, we found that RIP3–M45 hetero-fibrils formed clusters that were morphologically distinct from RIP3 fibrils in EM (Fig. 5d). In addition, AFM of diluted RIP3–M45 hetero-fibrils showed differences from RIP3 or M45 homo-fibrils. Unlike pure RIP3 fibrils, the head-tail interactions could rarely be detected in AFM of RIP3–M45 hetero-fibrils, and the RIP3–M45 heteroamyloid cluster consisted of a parallel arrangement of single fibrils with similar heights, unlike the fibrillar bundles with various heights of pure M45 fibrils (Figs. 2c and 5e). These results suggest that the free M45–RHIM peptides do not block RIP3 fibrillar polymerization in vitro but form M45–RIP3 hetero-fibrils, which may change the inter-filament architecture compared with pure RIP3 or M45 homo-fibrils. We then wanted to determine whether M45 homo- or M45–RIP3 hetero-fibrils inhibit RIP3–RHIM fibrillar polymerization. The ThT fluorescence increase of RIP3 peptides was measured when seeds of RIP3, M45, or M45–RIP3 amyloid polymer were present. These results show that the RIP3, M45, or M45–RIP3 amyloids could serve as nuclei to accelerate RIP3–RHIM formation of a homo-amyloid oligomer (Fig. S4A). It indicates that M45 amyloids did not inhibit but instead promoted RIP3–RHIM amyloid formation in vitro. Interestingly, the RIP3 amyloids could further assemble into large amyloid polymers in vitro, which were deformed in M45–RIP3 heteroamyloids (Fig. S4B). The in vitro data suggest that M45 does not affect RIP3 amyloid formation. We then tested whether M45 could affect RIP3 amyloids formation in cells. Most of amyloids are insoluble protein aggregates which are enriched in cell lysis pellet after high-speed centrifugation. Therefore, cellular RIP3 fibril formation could be detected by measuring RIP3 translocation to cell pellets upon necroptosis induction. We found that when necroptosis is induced, both RIP3 and M45 can translocate to this amyloid-enriched pellet fraction (Fig. 5f). This means that necroptosis stimuli could induce both RIP3 and M45 to form amyloids in cells. Thus, RIP3 puncta formation is blocked by M45 after RIP3 forms fibrils. The RIP3/M45 in pellet fraction are resistant to urea. It confirmed they form amyloids (Fig. S4C). We have shown that M45 could block RIP3 autophosphorylation (Fig. S2D). It means that RIP3 amyloid formation is required but not sufficient for kinase activation. Interestingly, previous studies showed that the kinase dead form of RIP3 could not form puncta upon necrosis induction [5]. It may be caused by the inactive kinase domain leading a close state conformation that mask RIP3–RHIM. So that if M45 could directly inhibit RIP3 kinase activity, it could block RIP3 puncta formation indirectly. By in vitro kinase assay, we confirmed that M45–RHIM could not directly inhibit RIP3 kinase activity (Fig. S4D). Therefore, M45 could block RIP3 puncta formation through directly interfering RIP3–RHIM amyloids further assembly, which was required for RIP3 kinase activation.

### RIP3 mutations impairing the inter-filament interactions disrupted necroptosis

**Fig. 6:**
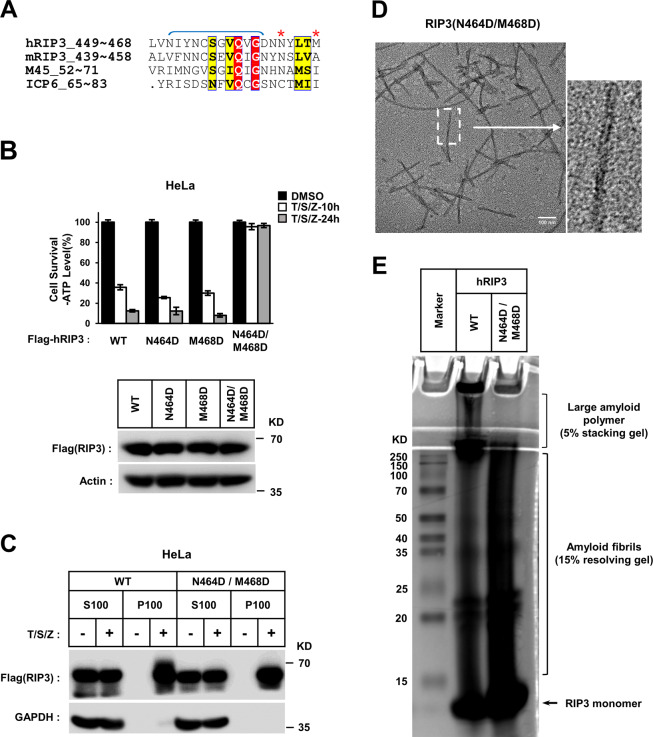
RIP3 mutations impairing the inter-filament interactions disrupted necroptosis. **a** Sequence alignment of RHIM domain from human RIP3, mouse RIP3, M45, and ICP6. The identical and conserved residues were highlighted in red and yellow, respectively. The N464 and M468 were indicated by red asterisk. The region in RIP3–RHIM involves in intra β-sheet interactions required for amyloid formation, revealed by the RIP1/3 amyloid structure (PDB code 5V7Z), was indicated by dark blue bracket. **b** Mutation of both Asn464 and Met468 in human RIP3 to aspartic acid led to complete inhibition of the TNF-induced necroptosis. The Hela cells with indicated lentivirus infection were treated T/S/Z for 10 and 24 h, respectively. The number of surviving cells was measured by measuring ATP levels (upper). The data are represented as the mean ± SD of duplicate wells. The RIP3 expression level was analyzed by western blot (lower). **c** Both wild-type and mutant (N464D/M468D) RIP3 will translocate to amyloid-enriched pellet fraction upon necrotic induction. The Hela cells with indicated lentivirus infection were treated with the indicated stimuli for 6 h. The cells were harvested and homogenized, then separated into the indicated fractions as described in “Materials and Methods.” These fractions were analyzed by western blotting using antibodies as indicated. S supernatant, P precipitate. **d** EM images of RIP3 (N464D/M468D) fibrils. The amyloid fibrils were prepared as described in “Materials and Methods.” Fibers were detected by TEM. Scale bar: 100 nm. **e** Comparison of inter-filament assembly between wild-type and mutant RIP3 fibers. The amyloid fibrils were prepared as described in “Materials and Methods.” Aliquots of 20 μg fresh prepared RIP3 and RIP3 (N464D/M468D) amyloid fibrils were analyzed by SDS-PAGE with Coomassie blue staining.

Our data suggested that RIP3 activation requires free RIP3 proteins to form amyloid fibrils and then self-assemble (properly through head-tail connections) into large fibrillar networks (RIP3 puncta). The latter step could be blocked by M45–RHIM amyloid incorporation. So that RIP3–RHIM took charge of not only amyloid formation but also inter-filament assembly. The region from N451 to D462 of RIP3 containing the four conserved residues (VQVG) was responsible for amyloid formation revealed by the RIP1/3 amyloid structure (PDB code 5V7Z) (Fig. 6a, indicated by dark blue bracket). We then suspected amino acids outside of this region may be responsible for inter-filament assembly and were critical for RIP3 mediated cell necroptosis. To identify the critical amino acid residues in RHIM core of RIP3, several RIP3 mutants have been tested. We found alanine mutations of eight residues L449, N451, Y453, N464, Y465, L466, T467, and M468 totally blocked necroptosis, while alanine mutations of six residues L449, N451, Y453, Y465, L466, and T467 only partially blocked necroptosis (Fig. S5A, B). It suggested that N464 and M468 of RIP3 were important in necroptosis signaling. We then mutated N464 and M468 to alanine or aspartic acid, and found that the N464A/M468A mutant was still functional to mediate cell necroptosis (Fig. S5C). But double mutation of N464 and M468 to aspartic acid (N464D/M468D) blocked cell necroptosis induced by TNF or IFN (Figs. 6b and S5D). Moreover, the necrosis signal could not induce activation of mutant RIP3, but the translocation of RIP3 to amyloid-enriched pellet fraction was not affected (Figs. S5E and 6c). It suggested that these mutations of RIP3 did not affect amyloids formation upon necrosis induction. Indeed, we found that RIP3_N464D/M468D_ mutant proteins could form amyloid fibrils in vitro, morphologically similar with wild-type RIP3 in EM (Fig. 6d). While in AFM, mutant RIP3 formed uniformly dispersed short fibrils, which were comparable to wild-type RIP3 fibrils (Fig. S5F). As in AFM of RIP3–M45 hetero-fibrils, the head-tail interactions of mutant RIP3 fibrils were rarely seen, suggesting that the mutant RIP3 reduced the proper ordered inter-filament interactions. Consisted with that, in vitro assembly assay showed a lot of wild-type RIP3 form ultra-large amyloid fibers in stacking gel, but only a few mutant RIP3 (N464D/M468D) signal was in there (Fig. 6e). It indicated the deficiency of mutant RIP3 to assemble into large amyloid polymer. We also compared necroptotic signal-induced self-assembly of wild-type and mutant RIP3 in cells, using chemical crosslinker disuccinimidyl glutarate. It showed that comparing with wild-type RIP3, the signal of assembled mutant RIP3 was reduced (Fig. S5G). All of the above suggested the mutation of N464/M468 to aspartic acid in RIP3 block necroptosis through interfering ordered inter-filament assembly of RIP3. Besides the human RIP3 mutant, a mouse RIP3 mutant (L456D) with the mutation site away from the tetrad core sequence of RHIM was also identified. The mutation blocked TNF- or IFN-induced RIP3 activation and cell necroptosis (Fig. S6A, B), which may also be due to interfere ordered inter-filament assembly. These data confirmed the importance of inter-filament mediated self-assemble of RIP3 in necroptosis signaling. Altogether, our data suggested a working model of RIP3 activation and M45 function (Fig. S6C). The amyloid fibril architectures of RIP3–RHIM and M45–RHIM differ. These two types of amyloid fibrils alone can further assemble to form a functional high-order amyloid network through ordered inter-filament interactions (Fig. S6C, a and c). It leads to RIP3 activation and cell necroptosis. Mutations impairing inter-filament interaction prevent RIP3 amyloids further assembly (Fig. S6C, a). However, when different types of RHIM are in the same cells, they will form neutralized hetero-fibrils to prevent higher-order amyloid network formation (Fig. S6C, b).

## Discussion

The RHIM of RIP1/3 can form A-beta-like amyloid fibrils in vitro and amyloid fibril formation is required for necroptosis signaling [5–7, 19]. Our data showed that RHIM-mediated amyloid formation of RIP3 and M45 is induced by an upstream necrosis signal, although RIP3 kinase activation and cell necrosis are blocked by M45 (Fig. 5f). Therefore, RIP3 amyloid formation is not sufficient for RIP3 activation. And we found that M45 blocks RIP3 function at the step of RIP3 amyloid fibril further assembly into cellular puncta. Previous studies found that the RHIM of RIP1, RIP3 or their hetero mixture could form amyloid fibrils with similar morphology by EM [18, 19]. However, compared to the RIP3 homo-RHIM fibrils, a significant change in the morphology of M45 homo- or M45–RIP3 hetero-fibrils was detected by EM in our study (Figs. 2a and 5d) [34]. Our AFM results suggest that the morphological differences may be caused by the distinct inter-filament interactions in RIP3, M45, and M45–RIP3 amyloids (Figs. 2c and 5e). This suggests that M45 prevents RIP3 fibrils from forming an amyloid network by disrupting ordered inter-filament interactions. Clearly, the core of the model is that M45–RHIM has a structure that is different from that of RIP3–RHIM. Although our EM and AFM data showed the different packing forms of RIP3 or M45 homo-fibrils and RIP3–M45 hetero-fibrils (Figs. 2a, c and 5d, e), further structural analysis to compare the M45 and RIP3 inter-filament architectures is still indispensable to reveal the detailed molecular mechanism by which M45 blocks RIP3 activation through hetero-RHIM-RHIM interactions. Furthermore, judging from their crystal structures, the architectures of the four conserved residues in the RHIM of RIP1 and RIP3 (IQIG of human RIP1 and VQVG of human RIP3) are almost the same [18, 19]. M45 has the same four conserved residues (IQIG) as RIP1 (Fig. S2B). Thus, the difference between the inter-filament architectures of M45–RHIM and RIP3–RHIM depends on another region of the RHIM, which is confirmed by our RIP3 mutant N464D/M468D. In addition to the M45–RIP3 hetero-RHIM polymer, a previous report also showed that the fibril morphology of the M45–RIP1 or M45–DAI RHIM mixture was different from that of the RIP1 or DAI homo-RHIM polymer by EM [34]. This suggests that M45 may also affect the inter-filament interactions of RIP1 or DAI. However, there is no evidence to suggest that RIP1 or DAI function requires inter-filament assembly of their RHIM amyloids. It will be interesting to analyze whether M45 can directly affect RIP1 or DAI function in subsequent studies, which may indicate whether the ordered inter-filament combination needed to form a network is generally required for the function of all RHIM-dependent amyloid proteins. It has been reported that the folding of RIP1/3-RHIM amyloid is structurally similar to that of the classical Aβ [19]. Plaque depositions of Aβ and other pathologic amyloids have been used as established biomarkers for Alzheimer’s disease, although the pathological roles of these amyloids are not fully understood. Our studies of RIP3/M45–RHIM function imply that the ordered inter-filament interactions of pathologic amyloids may contribute to amyloid deposition and disease progression, which may be a new therapeutic target for disease treatment.

## Supplementary information

Supplementary Figure Legends

Figure S1

Figure S2

Figure S3

Figure S4

Figure S5

Figure S6
