# Supplementary material for: RIP3-mediated necroptosis is regulated by inter-filament assembly of RIP homotypic interaction motif
Source: Cell Death Differ. 2020 Jul 31;28(1):251–66. doi: 10.1038/s41418-020-0598-9 (PMC7853141; doi:10.1038/s41418-020-0598-9)
Supplement: Supplementary file 1 — Supplementary Figure Legends [file 41418_2020_598_MOESM1_ESM.docx]

**Supplementary Figure Legends**

**Figure S1**

**A** Schematic representation of full-length ICP6 and ICP6 (1-280aa) with or without RHIM mutations used in these studies (left) and chimaeric M45 containing RIP3 or ICP6 RHIM (right). The four-conserved residues (VQCG of ICP6) of RHIM or their alanine mutations destroy RHIM function are indicated.

**B-C** Cell necroptosis is blocked by full-length or N-terminal portion of M45. The cells with indicated lentivirus infection were treated with T/S/Z for HeLa-RIP3 cells (B), or with T/Z for mouse fibroblast L929 cells (C) to induce necroptosis. The number of surviving cells with necrosis stimuli for 10 hours and were determined by measuring ATP levels using Cell Titer-Glo kit (lower). The M45 expression level was measured by western blot analysis (upper).

**D** Both RHIM and R1 domain of ICP6 were required for blockage of cell necrosis. The HT29 cells with indicated lentivirus infection were treated T/S/Z for 10 hours. The number of surviving cells was analyzed by measuring ATP levels (lower). The data are represented as the mean ± SD of duplicate wells. The ICP6 expression level was measured by western blot analysis (upper). Asterisk (*) denotes non-specific band.

**E** Induced RHIM oligomerization could not help to enhance the necroptosis blockage function of M45. HT29 cells stably expressing M45(1-292)_2FKBPv were treated with indicated stimuli. The number of surviving cells was determined (lower). Data are representative of the mean ± SD of duplicate wells. The M45(1-292)_2FKBPv expression level was measured by western blot analysis (upper).

**Figure S2**

**A** Schematic representation of M45 truncations.

**B** Sequence alignment of RHIM core region from RIP1, RIP3, M45 and ICP6. The four-conserved residues were highlighted in bold. The corresponding sequences of RHIM core were swapped in chimaeric RIP1/3 and chimaeric M45 for these studies.

**C** Mapping the M45 functional region. HT29 cells were infected with viruses encoding truncated M45 (indicated in A), then treated with T/S/Z for the indicated time. The number of surviving cells was determined (lower). The whole-cell lysates were subjected to SDS-PAGE and measured by western blot analysis with the indicated antibodies (upper). Statistical significance was determined using two-way ANOVA followed by a Dunnett post-hoc test; * P < 0.05 and *** P < 0.001.

**D** M45 RHIM could block RIP3 activation. HT29 cells containing truncated wild-type M45(1-292) or M45(1-90) were treated with T/S/Z for 8 hours. Then the cells were lysed and analyzed by western blot using p-RIP3 antibody to indicate RIP3 activation.

**E** The chimaeric M45 containing RHIM of ICP6 or RIP3 could not block RIP3 activation. HT29 cells containing indicated chimaeric M45 were treated with T/S/Z for 8 hours. RIP3 activation was analyzed by western blot using p-RIP3 antibody.

**F** The chimaeric M45 containing RHIM of ICP6 or RIP3 could retain necrosis process. HeLa-RIP3 cells with indicated lentivirus infection were treated with T/S/Z for indicated time. The number of surviving cells was determined by measuring ATP levels (lower). The data are represented as the mean ± SD of duplicate wells. The expression level of chimaeric M45 was measured by western blot analysis (upper). Statistical significance was determined using two-way ANOVA followed by a Dunnett post-hoc test; * P < 0.05, ** P < 0.01.

**Figure S3**

**A** Schematic representation of chimaeric RIP3 containing M45/ICP6 RHIM (left) or M45 N- terminus (right).

**B-C** RIP3 chimeras with M45 N-terminus could restore the sensitivity to necroptosis induction. HeLa (B) or NIH-3T3 (C) cells with indicated lentivirus infection were treated with T/S/Z or AP20187 for 10 hours. The number of surviving cells was determined by measuring ATP levels (lower). The data are represented as the mean ± SD of duplicate wells. The chimaeric RIP3 expression level was measured by western blot analysis (upper).

**D** NIH-3T3_2FKBP_V_-mRIP3 cells with indicated virus infection were treated with AP20187 for indicated time. The number of surviving cells was determined by measuring ATP levels. Data are represented as mean ± SD of duplicate wells. Statistical significance was determined using two-way ANOVA followed by a Tukey post-hoc test; * P < 0.05, **P < 0.01 and ***P < 0.001.

**E-F** RIP3-M45 RHIM hetero complex can block RIP3 function. HeLa (E) cells with expression of hRIP3 or chimaeric hRIP3_M45_RHIM_ and NIH-3T3 (F) cells with expression of tandem FKBPV fused mRIP3 or chimaeric mRIP3_M45_RHIM_ were infected with empty viruses (Vector) or lentiviruses encoding M45(1-292)_RIP3_RHIM_. The cells were treated as indicated for 10 hours. The number of surviving cells was determined by measuring ATP levels (lower). The data are represented as the mean ± SD of duplicate wells. The M45/RIP3 chimera expression level was measured by western blot analysis (upper). Asterisk (*) denotes non-specific band.

**Figure S4**

**A** Cross-seeding of RIP3 amyloid formation with indicated seeds monitored by ThT fluorescence binding assay. Denatured human RIP3 peptides were diluted to a final concentration of 10 μM with ThT as described in Methods. The cross-seeding was performed by adding 1 μM seeds.

**B** The RIP3 amyloids could further assemble to large amyloid polymers in vitro, which were deformed in M45-RIP3 hetero amyloids. The amyloid fibrils were prepared as described in Methods. Aliquots of 20 μg fresh prepared RIP3 and RIP3-M45 amyloid fibrils were subjected to SDS-PAGE followed by Coomassie blue staining analysis.

**C** Stability assessment of the endogenous M45/RIP3 aggregation. HT-29 cells with or without M45 expression were treated with the indicated stimuli for 8 hours. The cells were harvested and homogenized, then separated into the indicated fractions as described in the Methods. These fractions were analyzed by western blotting using antibodies as indicated. S, supernatant; P, precipitate.

**D** In vitro kinase assay. RIP3 and M45 were immunopurified by anti-Flag beads, and used in a kinase assay as described in Methods.

**Figure S5**

**A** Schematic representation of wild type and mutant human RIP3. The RIP3 sequences from Leu449 to Met468 were listed and the alanine mutations were indicated. The four conserved residues (VQVG) were highlighted in green. The Asn464 and Met468 were highlighted in yellow.

**B** Asn464 and Met468 were important to RIP3 function. The Hela cells with indicated lentivirus infection were treated with T/S/Z for 24 hours. Cell viability was determined by measuring ATP levels (upper). The data are represented as the mean ± SD of duplicate wells. The expression level of RIP3 was measured by western blot analysis (lower).

**C** Alanine mutations of N464 and/or M468 have no effect on TNF-induced necroptosis. The Hela cells with indicated lentivirus infection were treated with T/S/Z for 24 hours. Cell viability was determined by measuring ATP levels (upper). The data are represented as the mean ± SD of duplicate wells. The expression level of RIP3 was measured by western blot analysis (lower).

**D** Mutation of both Asn464 and Met468 in human RIP3 to Asp led to complete inhibition of the IFN-induced necroptosis. The Hela cells with indicated lentivirus infection were treated as indicated for 24 hours. IFN: Interferon-gamma (100 ng/ml). The number of surviving cells was measured by measuring ATP levels (upper). The data are represented as the mean ± SD of duplicate wells.

**E** RIP3 and MLKL could not be activated in the HeLa cell expressing RIP3 (N464D/M468D) upon necroptosis induction. Cell necroptosis was induced by T/S/Z for 8 hours. Then the cells were lysed and subjected to western blotting analysis using the p-RIP3 or p-MLKL antibodies to indicate RIP3 or MLKL activation.

**F** AFM images and the height profile of RIP3 (N464D/M468D) fibrils. The height profile data was obtained from the locations indicated by these arrows. It shows that the average height of the fibers is 2.9 ± 0.3nm.

**G** Detection of self-assembly of wild-type or mutant RIP3(N464D/M468D) using chemical crosslinker disuccinimidyl glutarate (DSG) after necroptosis induction. HeLa cells with Flag-tagged wild-type or mutant RIP3(N464D/M468D) expression were treated with DMSO or T/S/Z for 10 hours. The cellular RIP3 were immunopurified by incubation with anti-Flag magnetic beads (Bimake) at 4 ℃ overnight. The next day, beads were washed 4 times with Phosphate-buffered saline(PBS), then DSG was added to the sample at a final concentration of 5 mM. The crosslinking reaction was performed in PBS at room temperature for 30 minutes and stopped with buffer containing 1 M Tris. The samples were analyzed by western blotting using the M2-Flag antibody.

**Figure S6**

**A-B** Mutation of Leu456 in mouse RIP3 to Asp led to complete inhibition of the TNF-induced (A) or IFN-induced (B) necroptosis. NIH-3T3 cells infected with lentivirus containing wild-type or mutant RIP3 (L456D) were stimulated with TNF-α(10 ng/ml) /Smac mimetic/z-VAD-fmk for 10 hours (A) or IFN(100 ng/ml)/Smac mimetic/z-VAD-fmk for 24 hours (B). Cell viability was determined by measuring ATP levels.

**C** Schematic model of RHIM amyloid dependent RIP3 activation and M45 inhibition. The RIP3, M45 and RIP3-M45 homo- or hetero- RHIM fibrils are present as yellow camber (a), green rectangle (c), and green and yellow cylinder (b) respectively, which indicates the different architectures. The mutations impairing the inter-filament interaction were indicated by red asterisks. (a) the RIP3 fibrils could assembly to large complex through inter-fibril interactions which is require for RIP3 puncta formation, kinase activation and cell necrosis. Mutations of RIP3 impairing the inter-filament interactions prevent RIP3 fibrils assembly; (b) the M45 binds to RIP3 which form hetero fibrils which structurally change the RIP3 fibrils. It will prevent self-pile-up of RIP3 to form high-order polymer; (c) the M45 RHIM fibril could also have the ability to form high-order polymer. It could be functional when embedded to RIP3_M45_RHIM_, and blocked by M45_RIP3_RHIM_ through M45-RIP3 hetero fibrils (indicated in b).
