# Supplementary figures and images for: RIP3-mediated necroptosis is regulated by inter-filament assembly of RIP homotypic interaction motif
Source: Cell Death Differ. 2020 Jul 31;28(1):251–66. doi: 10.1038/s41418-020-0598-9 (PMC7853141; doi:10.1038/s41418-020-0598-9)

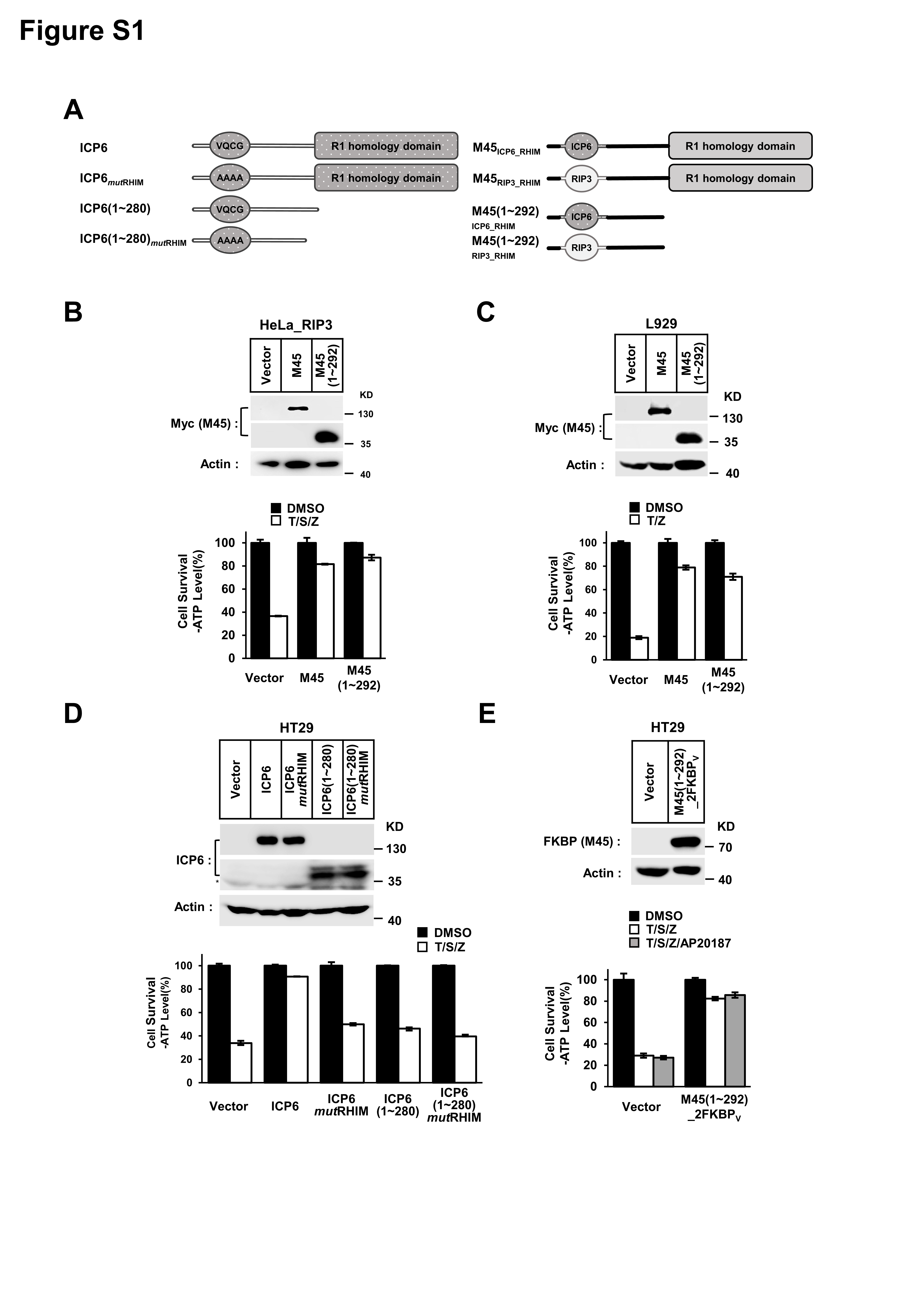

Supplement: Supplementary file 2 — Figure S1 [file 41418_2020_598_MOESM2_ESM.png]

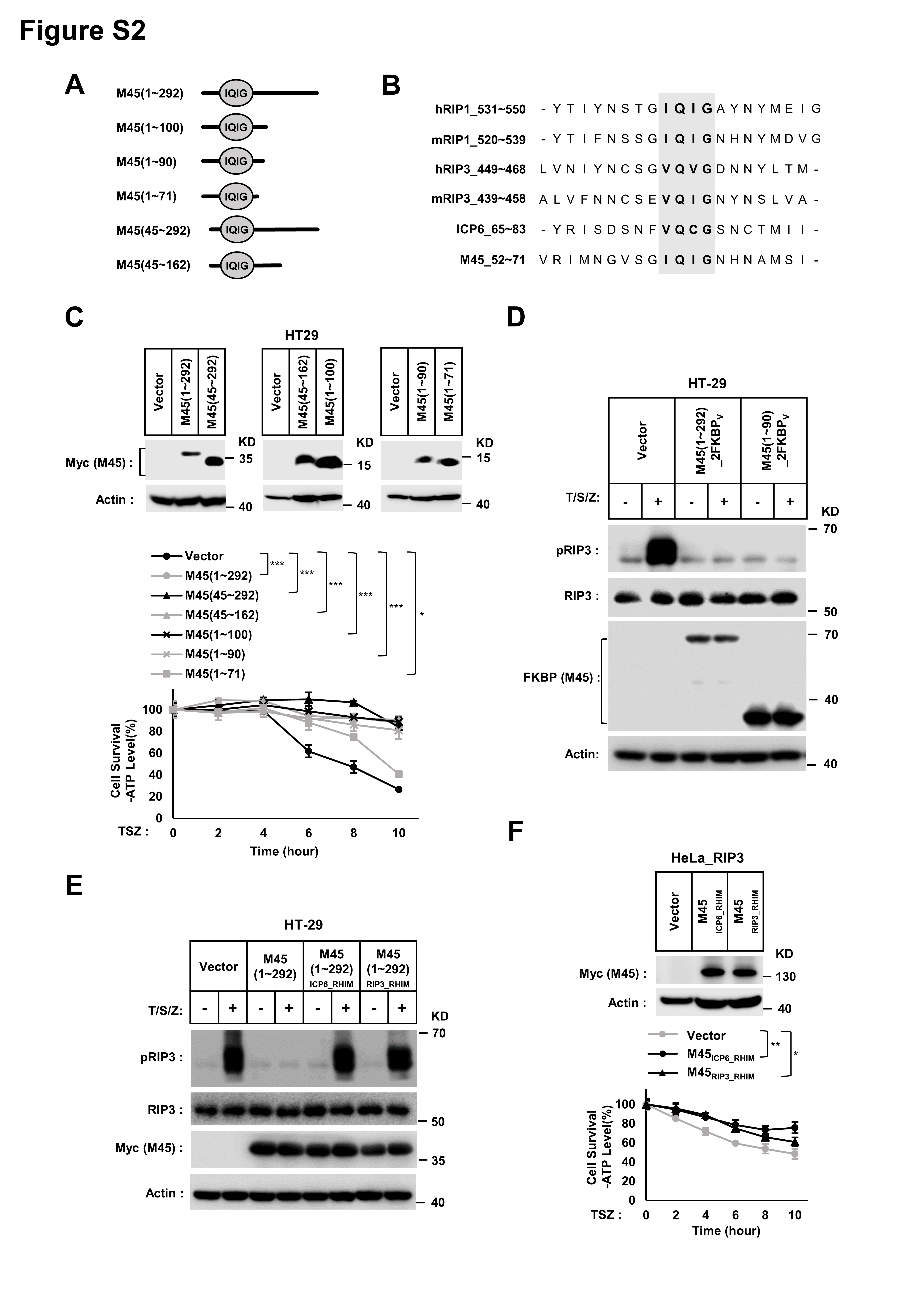

Supplement: Supplementary file 3 — Figure S2 [file 41418_2020_598_MOESM3_ESM.png]

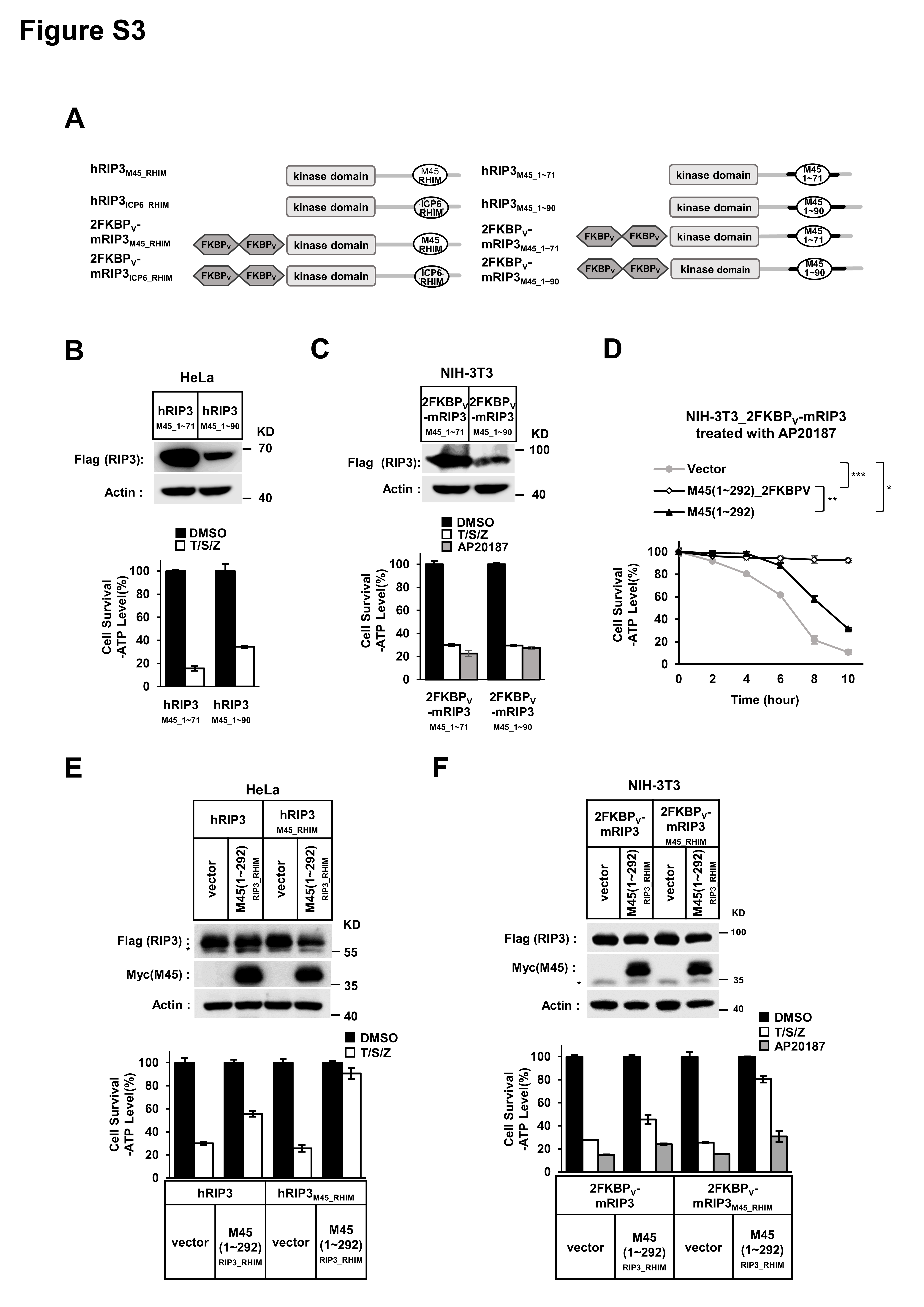

Supplement: Supplementary file 4 — Figure S3 [file 41418_2020_598_MOESM4_ESM.png]

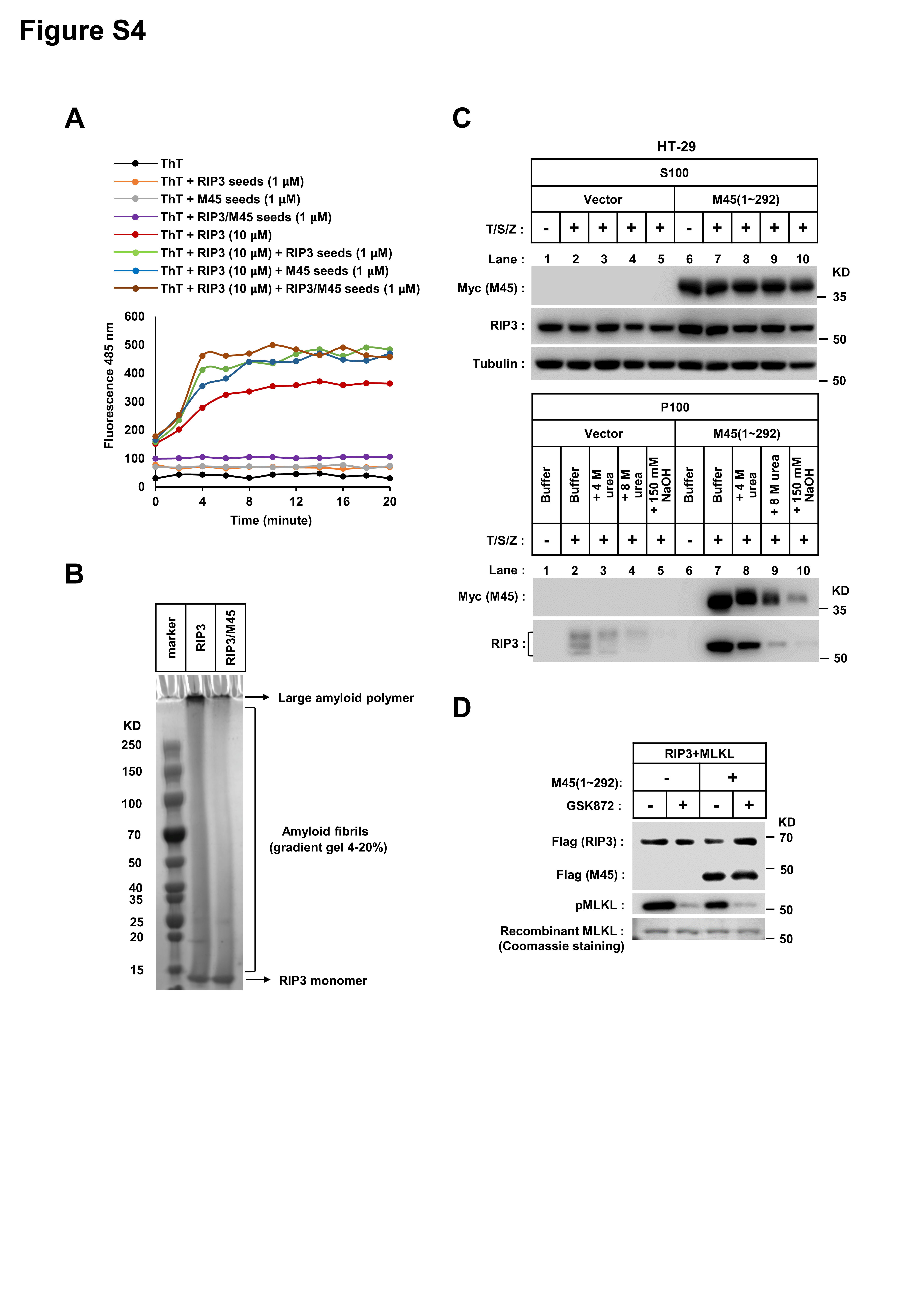

Supplement: Supplementary file 5 — Figure S4 [file 41418_2020_598_MOESM5_ESM.png]

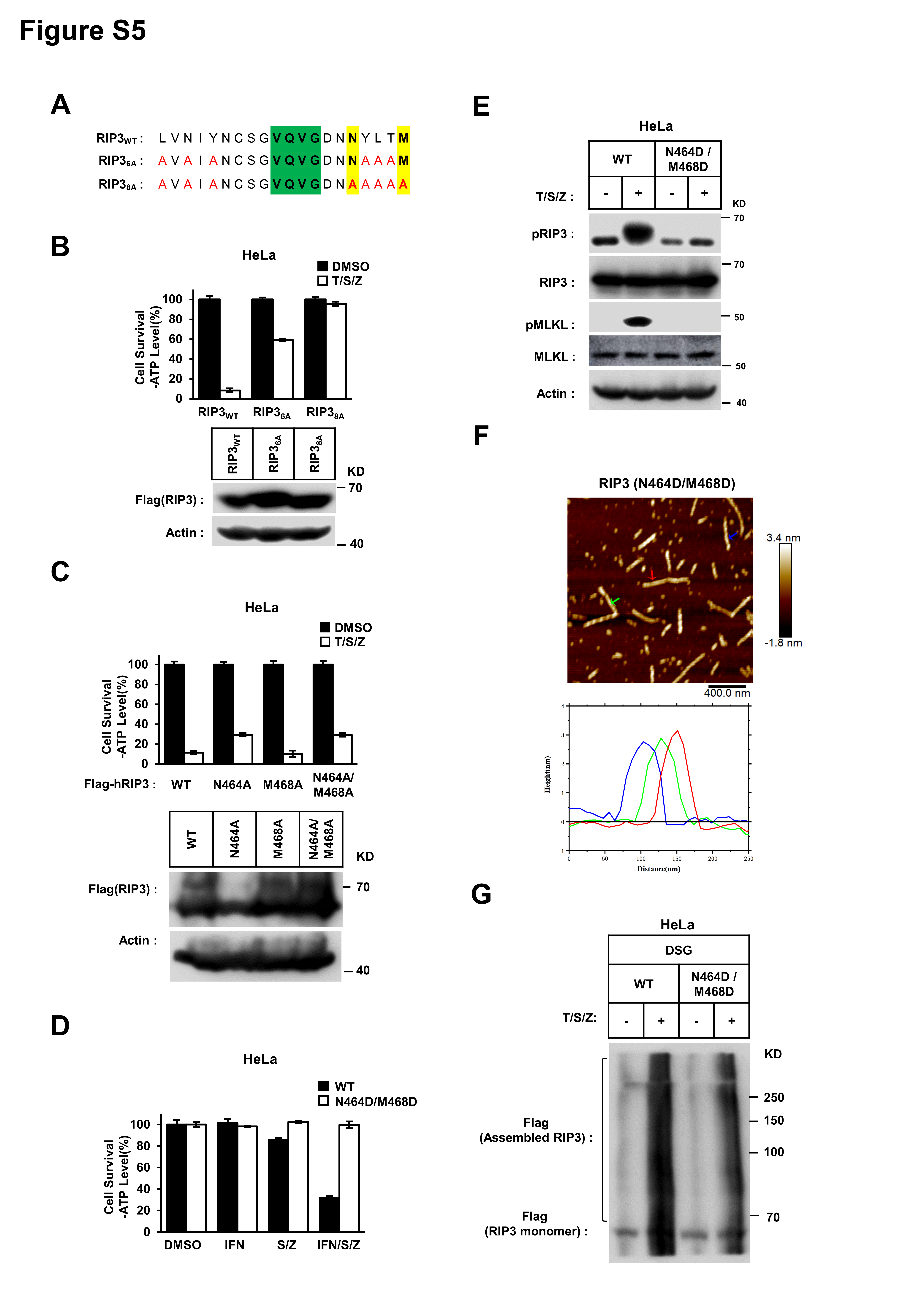

Supplement: Supplementary file 6 — Figure S5 [file 41418_2020_598_MOESM6_ESM.png]

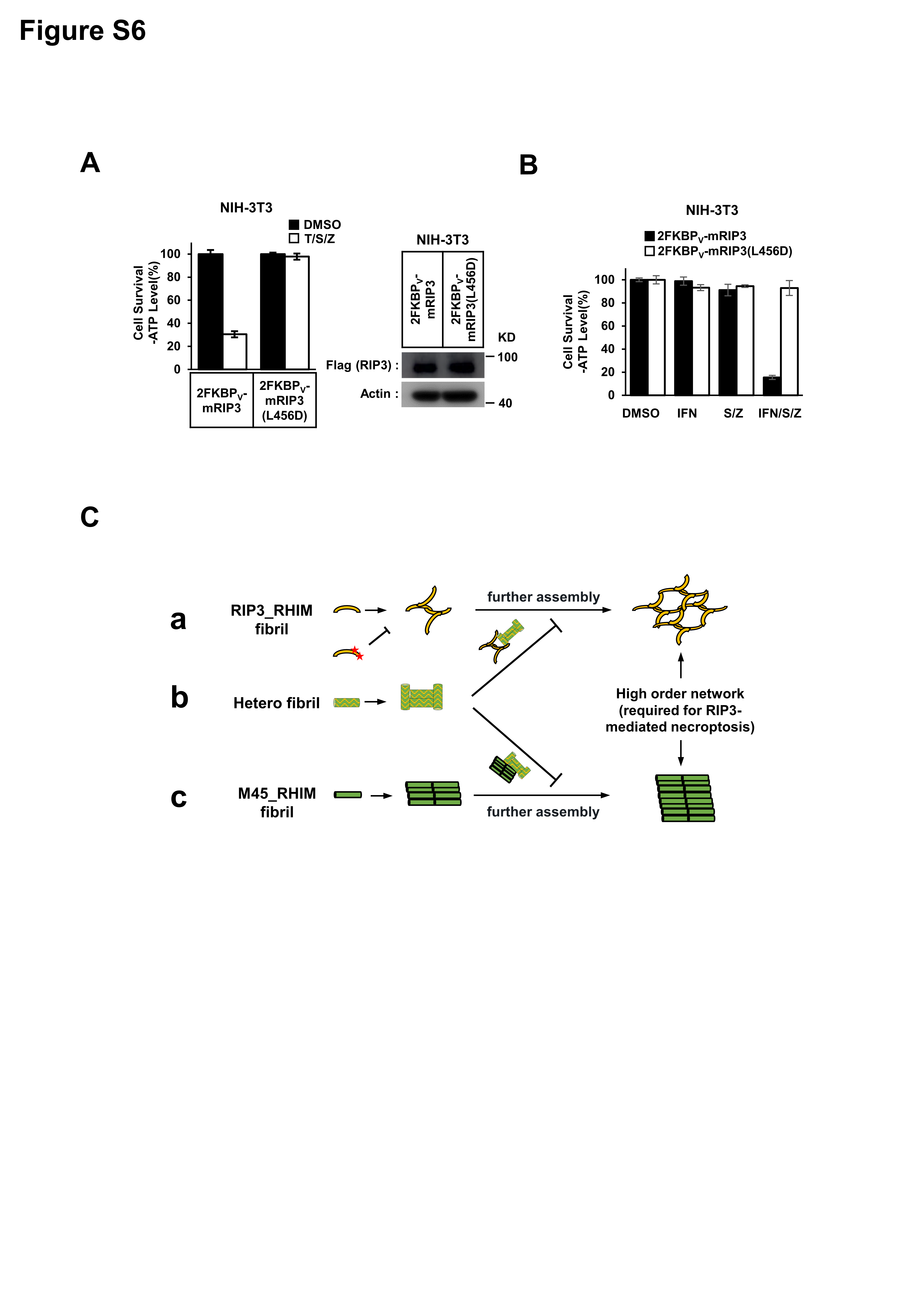

Supplement: Supplementary file 7 — Figure S6 [file 41418_2020_598_MOESM7_ESM.png]
